# High Infection Risk Among Health Care Workers During the First SARS‐CoV‐2 Wave in Niamey, Niger

**DOI:** 10.1111/irv.70177

**Published:** 2025-10-25

**Authors:** Hamidou Lazoumar Ramatoulaye, Aliou Sanda Abdal‐Kader, Adamou Lagare, Mahamadou Almahamoudou Maiga, Fakani Aboutalib Aliane, François Comlan Aida Sylviane, Idi Issa, Bibata Abdou Sidikou, Garda Idé Oumarou, Ali Sidiki, Zeinabou Abdou Aouta, Amina Moussa, Zeinabou Dioffo Alassan, Ibrahim Karidio, Goni Alhassan Maman Bachir, Issifou Djibo, Salia Moussa, Ibrahim Maman Laminou, Ronan Jambou, Vincent Richard

**Affiliations:** ^1^ Centre de Recherche Médicale et Sanitaire Niamey Niger; ^2^ Faculté des Sciences et Techniques de l'Université Abdou Moumouni de Niamey Niamey Niger; ^3^ EPICENTRE Niamey Niger; ^4^ Faculté des Sciences de la Santé de l'Université André Salifou de Zinder Hôpital Zinder Niger; ^5^ Direction de la Surveillance et de la Riposte aux Épidémies Niamey Niger; ^6^ Hôpital Général de Référence Niamey Niger; ^7^ Institut Pasteur de Paris, France Paris France

**Keywords:** COVID‐19, health workers, Niger, risk factors

## Abstract

**Background:**

In 2020, the new pathogen SARS‐CoV‐2 spread fast, causing a pandemic. Health care workers on the frontline were of course highly exposed. This study aims to analyze the risk factors of SARS‐CoV‐2 infection in HCWs who have been in contact with positive patients in Niger.

**Methods:**

A prospective cohort was conducted among HCWs from March 2020 to June 2020 in health facilities in Niamey. A questionnaire was administered at inclusion; RT‐PCR testing was performed if clinical signs were present. Serological testing was performed at baseline, Days 15 and 30. Univariate analysis and Cox regression were used.

**Results:**

Regarding inclusion criteria, 129 health care workers were included. The sex ratio (male/female) was 0.82. The participants were mainly physicians (45.7%) and nurses (34.1%). At inclusion, the prevalence of COVID‐19 was 34.9%. Only seronegative (*n* = 84) were followed up; the attack incidence rate for the first month was 440 per 1000 person*month. Regarding the Cox model, the use of alcohol‐based hand washing was a protective factor (RR = 0.28, *p* = 0.01). Furthermore, females were more at risk than males (RR = 2, *p*‐value = 0.049).

**Conclusions:**

HCWs in Niger were faced with high infection risk; this should lead decision‐makers to (i) enhance training on preventive measures and (ii) boost access to personal protective equipment in emergency and infectious disease wards.

## 1 Introduction

The COVID‐19 outbreak first detected in the city of Wuhan, China, in December 2019 [1], has caused a global health crisis, despite the implementation of large‐scale control measures. It rapidly spread across the different continents of the world. On January 30, 2020, the World Health Organization announced the outbreak of the new coronavirus, SARS‐CoV‐2, as a public health emergency of international concern, declaring it a pandemic on March 11, 2020 [2].

The African continent was the last to be affected after Europe and the United States. Africa recorded its first case officially on February 15, 2020, 2 months after it was first identified in China [3]. The first confirmed case of COVID‐19 in Africa was reported in Egypt on February 14 and the second one a day later in Algeria. By March 2020, COVID‐19 cases were reported from most of the continent. By April, nearly every country in Africa had reported COVID‐19, with hundreds, if not thousands, of cases reported in the hardest‐hit countries [4]. Niger had implemented border surveillance, mainly at the airport. The country had been facing the COVID‐19 pandemic since March 19, 2020, when the first case was reported. All of the eight regions of the country are affected by the pandemic [5]. According to the situation report of WHO, the virus was first reported in Niamey, before spreading to other parts of Niger [6].

Faced with this COVID‐19 pandemic, professionals working in health facilities were particularly exposed and so vulnerable to contamination. They were at the forefront of the epidemic, at higher risk of contamination and subsequent transmission to new patients, colleagues, and family [7]. In Africa, despite regular confrontation to epidemics [8], they faced several challenges in the context of coronavirus disease, such as lack of personal protective equipment (PPE), infection, quarantine, discrimination, and attacks on them in society, at the same time as a large responsibility in taking care for their relatives and families [9]. In some areas, health care workers had accounted for up to 11% of all confirmed COVID‐19 cases, with an increasing number of work‐related deaths [10].

In the countries with limited resources like Niger, it was thus crucial to determine which practices were the most effective to prevent contamination of health care workers, to subsequently focus on their dissemination.

In Niger, neither the spread of the first cases of SARS‐CoV‐2 infection in health care settings nor the analysis of risk factors associated with this infection specific to health care workers was clearly documented. This study was initiated to observe the spread of infection and to identify the risk factors for SARS‐CoV‐2 among health care workers. The main focus was the health units responsible for managing the first COVID‐19 cases.

## 2 Methods

### 2.1 Study Design

A prospective cohort study was conducted from March 20, 2020 to June 30, 2020 among people working in health facilities of Niamey where first cases of COVID‐19 were reported on March 19 2020. The recruitment of participants took place between March 2020 and May 2020.

### 2.2 Site of Study

Both private and public structures were considered as soon as they were in charge of COVID‐19 cases. Data were collected in 10 health facilities (Table 7): five hospitals (General Reference Hospital, National Hospital of Niamey, Issaka Gazobi Maternity, University Hospital Center, Regional Hospital Center, and Medical and Health Research Center [reference laboratory]) and four health care centers (Mamar Kassey Clinic, Ambulatory Treatment Center, Gawaye Health Center, and Arahama Health Center).

### 2.3 Study Population and Follow‐Up Procedure

In this study, all health care workers in charge of SARS‐CoV‐2 infected patients and with a contact less than 15 days were eligible, whatever their positions, including those who handled blood samples or were in contact with biological fluids through cleaning of the surfaces or equipment.

The health workers who agreed to participate were included in the study and were followed for 1 month during which they were called every day to inquire about their health status (“presence or absence of clinical signs in favor of SARS‐CoV‐2 disease, temperature measurement”). All people presenting clinical signs during the month of follow‐up were tested by PCR for SARS‐CoV‐2 detection.

### 2.4 Data and Samples |Collected

At inclusion, a standard questionnaire was completed including information on sociodemographic characteristics, exposure to the SARS‐CoV‐2 infected patient (number of contacts, time of contact, type of contact, etc.), and personal and collective infection prevention facilities. Furthermore, blood samples of 2–3 mL were taken at Day 0.

For the follow‐up step, only seronegative HCWs at enrolment were invited to participate. Blood samples of 2–3 mL were suggested and collected on Days 14 and 30. In addition, in case of clinical signs (Table 1), nasopharyngeal samples were proposed for biological diagnosis.

**TABLE 1:** Sociodemographic characterization of the population at inclusion (*N* = 129).

Characteristics	Total (*N* = 129)		Seronegative (*N* = 84)		Seropositive (*N* = 45)		*p*‐value
Age (median, IQR)	35	[30–43]	34.5	[30–43]	36	[30–43]	0.66
Age group	***N***	**(%)**	***N***	**(%)**	***N***	**(%)**	
(20–30 years)	43	(33)	30	(70)	13	(30)	0.51
(30–40 years)	46	(36)	27	(59)	19	(41)	
More than 40 years	40	(31)	27	(68)	13	(32)	
Gender							
Female	71	(45)	45	(63)	26	(37)	0.71
Male	57	(55)	39	(67)	19	(33)	
Professional status							
Physicians	59	(46)	41	(69)	28	(31)	0.55
Nurses	44	(34)	28	(64)	16	(36)	
Others	26	(20)	15	(58)	11	(42)	

Subjects were asked to provide verbal information about their use and practice of preventive measures against COVID‐19.

### 2.5 Biological Analysis

The nasopharyngeal samples were analyzed by RT‐PCR. The blood samples for serology were treated using WANTAI SARS‐CoV‐2 Ab ELISA (total antibodies Wantai Biological Pharmacy Enterprise Co. Ltd., Beijing, China).

The laboratory staff in charge of these different samples had been trained in the safe handling of samples and the implementation of virological confirmation of suspected cases of SARS‐CoV‐2.

### 2.6 Statistical Analysis

Data were collected on a database and analyzed with R software (R‐4.2.1).

For inclusion data, a general descriptive analysis of risk factors for SARS‐CoV2 infection among HCWs was first performed, and a Chi‐square or Fisher test was performed to compare proportions and a Test de Student for quantitative variables, considering a 5% margin of error.

For follow‐up data, RT‐PCR positivity or a serology positivity were considered as end point for the survival analysis. Kaplan–Meier methods were performed to estimate probabilities of COVID‐19 infection in HCWs, and the log‐rank test was conducted to assess the significance of differences in survival curves between groups and risk factor.

The survival analysis was extended using the Cox proportional hazard model to estimate the likelihood of SARS‐CoV‐2 infection and simultaneously assess the effect of several risk factors with a *p*‐value = 0.20 regarding Kaplan–Meier, adjusting for confounding or effect modification. A top‐down selection was performed, with the threshold determined by the Akaike information criterion (AIC). The conditions of realization of the Cox test were carried out; the assumption of proportionality was examined using statistical tests and graphical diagnostics based on the *scaled Schoenfeld residuals*.

### 2.7 Ethical Consideration

Ethical clearance was obtained from the National Ethics Committee for Health Research (CNERS) with the reference N°04/2020/CNERS during its session on March 12, 2020. Written informed consent was obtained by each participant. Respondents were informed that they had the right to refuse or discontinue their participation in the study at any time. The information collected was kept confidential. To this end, a code was assigned to each health facility, as well as an identification number for all study participants.

## 3 Results

### 3.1 Characteristics of HCWs at Inclusion

A total of 288 HCWs were contacted regarding inclusion criteria (less than 15 days after contact with a COVID‐19 patient) and consent to participate; 129 were included.

At inclusion (Table 1), the sociodemographic status revealed that the median age of the participants was 35 years. The sex ratio (M/F) was 0.8. According to the status, physicians were the most represented (46%), followed by nurses (34%) (Table 1).

Considering the serological status, 45 (35%) of the 129 included HCW were seropositive. No statistical differences were found considering the sociodemographic characteristics (Table 1).

Regarding the HCW's medical history (Table 2), 75% had declared no medical history, and 18% had only one comorbidity. Asthma (9%) and obesity (9%) were the most recorded risk factors.

**TABLE 2:** HCW's medical history.

	Total (*N* = 129)		Seronegative (*N* = 84)		Seropositive (*N* = 45)		*p*‐value
Clinical signs							
Asthenia	17	(13)	13	(15)	4	(9)	0,43
Cough	15	(12)	9	(11)	6	(13)	0,69
Sore throat	15	(12)	9	(11)	6	(13)	0,69
Headache	15	(12)	10	(12)	5	(11)	0.99
Myalgia	11	(9)	9	(11)	2	(4)	0,32
Runny nose	10	(8)	7	(8)	3	(7)	0,69
Fever	6	(5)	3	(4)	3	(7)	0,63
Ageusia	5	(4)	4	(5)	1	(2)	0,81
Loss of appetite	5	(4)	3	(4)	2	(4)	0.99
Anosmia	4	(3)	3	(4)	1	(2)	0.99
Dyspnea	3	(2)	3	(4)	0	(0)	0,28
Shivering	3	(2)	2	(2)	1	(2)	0.99
Medical history							
Obesity	12	(9)	7	(8)	5	(11)	0.84
Diabetes	3	(2)	3	(4)	0	(0)	0.49
Heart disease	4	(3)	3	(4)	1	(2)	0.99
Asthma	11	(9)	8	(10)	3	(7)	0.82
Chronic lung disease	2	(2)	1	(1)	1	(2)	0.99
Chronic hepatic disease	1	(1)	0	(0)	1	(2)	0.75
Chronic hematologic disease	3	(2)	3	(4)	0	(0)	0.50
Pregnancy (*N* = 71)	5	(7)	3	(7)	2	(7)	0.99
Chronic neurologic disease	1	(1)	0	(0)	1	(1)	0.75
Number of comorbidities							
No comorbidity	97	(75)	63	(75)	34	(76)	0.99
One comorbidity	23	(18)	15	(18)	8	(18)	
More than one	9	(7)	6	(7)	3	(7)	

Considering the serological status, no significant differences were found considering the medical history or clinical symptoms regarding the serological status.

In regard to the preventive measure (Table 3), the majority of the HCWs declared applying the preventive measures and protective recommendations. No statistical differences were found between serologically negative and serologically positive HCWs.

**TABLE 3:** Preventive and protective measures.

Characteristics	Total (*N* = 129)		Seronegative (*N* = 84)		Seropositive (*N* = 45)		*p*‐value
	*N*	(%)	*N*	(%)	*N*	(%)	
Training on hygiene and prevention measures	92	(71)	59	(70)	33	(73)	0.87
Follow recommended hand hygiene practices	127	(98)	82	(98)	45	(100)	0.76
Use alcohol‐based hand wash for hand hygiene	118	(91)	76	(90)	42	(93)	0.82
Use soap and water for hand hygiene	128	(99)	84	(100)	44	(98)	0.75
Hand washing before touching a patient	94	(73)	61	(27)	33	(27)	0.99
Hand washing before cleansing or asepsis	122	(95)	80	(95)	42	(93)	0.96
Hand washing after exposure (risk) to liquids	122	(95)	79	(94)	43	(96)	0.99
Hand washing after touching a patient	123	(95)	79	(94)	44	(98)	0.60
Hand washing after touching a patient's environment	112	(87)	69	(82)	43	(96)	0.06
Follow infection prevention and control precautions	121	(94)	79	(94)	42	(93)	0.99
Wear personal protection equipment when indicated	121	(94)	81	(96)	40	(89)	0.19

The majority of health care workers (65%) had a single contact with a first positive case of COVID‐19, a minority (3%) had two contacts, and 18% had three contacts. Fifty‐three percent of health care workers had contact times of less than 5 min, 32% between 5 and 15 min, and 15% more than 15 min. It should be noted that 42% of face‐to‐face contacts lasted less than 15 min, while 58% lasted more than 15 min. One percent of cases occurred during an aerosolization session. Ninety‐five percent of individuals observed hand hygiene before and after contact. Good hand hygiene is a key factor in reducing the transmission of infections. Eighty‐seven percent of individuals directly touched surfaces around the COVID‐19 positive patient (Table 4).

**TABLE 4:** Measurements of exposure of health care workers to SARS‐CoV‐2.

Measurements of exposure	Number	Percent (%)
Number of contacts		
1	84	65
2	4	3
3	23	18
ND	18	14
Contact time (min)		
< 5	69	53
5–15	41	32
> 15	19	15
Face‐to‐face contact		
< 15 mn	54	42
> 15 mn	75	58
Presence aerosolization session	1	1
Hand hygiene before contact	122	95
Hand hygiene after contact	122	95
Direct contact with surfaces around the patient	112	87

### 3.2 Follow‐Up Step

From the 129 included HCWs, 84 were seronegative at inclusion and were followed up. The sociodemographic status (Table 1) revealed that the median age of the participants was 34.5 years. The sex ratio (M/F) was 0.9. According to the status, physicians were the most represented (49%) followed by nurses (33%). No statistical differences were found for sociodemographic characteristics compared to seropositive HCWs.

Of the 84 HCWs followed up, 31 (37%) were COVID‐19 confirmed: 14 (45%) were both RT‐PCR positive and seropositive, 10 (32%) were RT‐PCR positive only, and 7 (23%) were seropositive only.

All of the 35 HCWs who presented with clinical symptoms during this 1‐month follow‐up period were collected for testing by RT‐PCR and serology for the diagnosis of COVID‐19, respectively. The sex ratio (M/F) was 0.75 (20 females [57%], 15 males [43%]). According to the status, physicians were the most represented (57%) followed by nurses (23%). Among these symptomatic HCWs, 15 (31%) were positive: 4 (27%) both RT‐PCR positive and seropositive, 7 (46%) were only RT‐PCR positive, and 4 (27%) were only seropositive.

Among the COVID‐19 symptomatic confirmed cases (*n* = 15), 8 (73%) were female and 7 (27%) were male. No statistical differences were found according to gender (*p*‐value = 0.39) or professional status (*p*‐value = 0.91): 30% of the symptomatic physicians confirmed, 38% of the nurses, and 29% of other regrouped categories.

Considering the clinical signs (Table 5), respiratory symptoms and headache were statistically associated with COVID‐19 confirmed cases (*p*‐value < 0.001).

**TABLE 5:** Comparison of the clinical symptoms among symptomatic HCWs regarding RT‐PCR results.

	Total (*N* = 35)		SARS‐CoV2 negative (*N* = 24)		SARS‐CoV2 positive (*N* = 11)		*p*‐value
	*N*	(%)	*N*	(%)	*N*	(%)	
Respiratory symptoms (*n* = 33)	7	(21)	3	(14)	4	(36)	< 0.001
Headache (*n* = 33)	3	(9)	0	(0)	3	(27)	< 0.001
Hospitalization	7	(20)	0	(0)	7	(64)	< 0.001

Obviously, hospitalization (*N* = 7) concerned 64% of the COVID‐19 confirmed cases but none of the negative cases.

**FIGURE 1:**
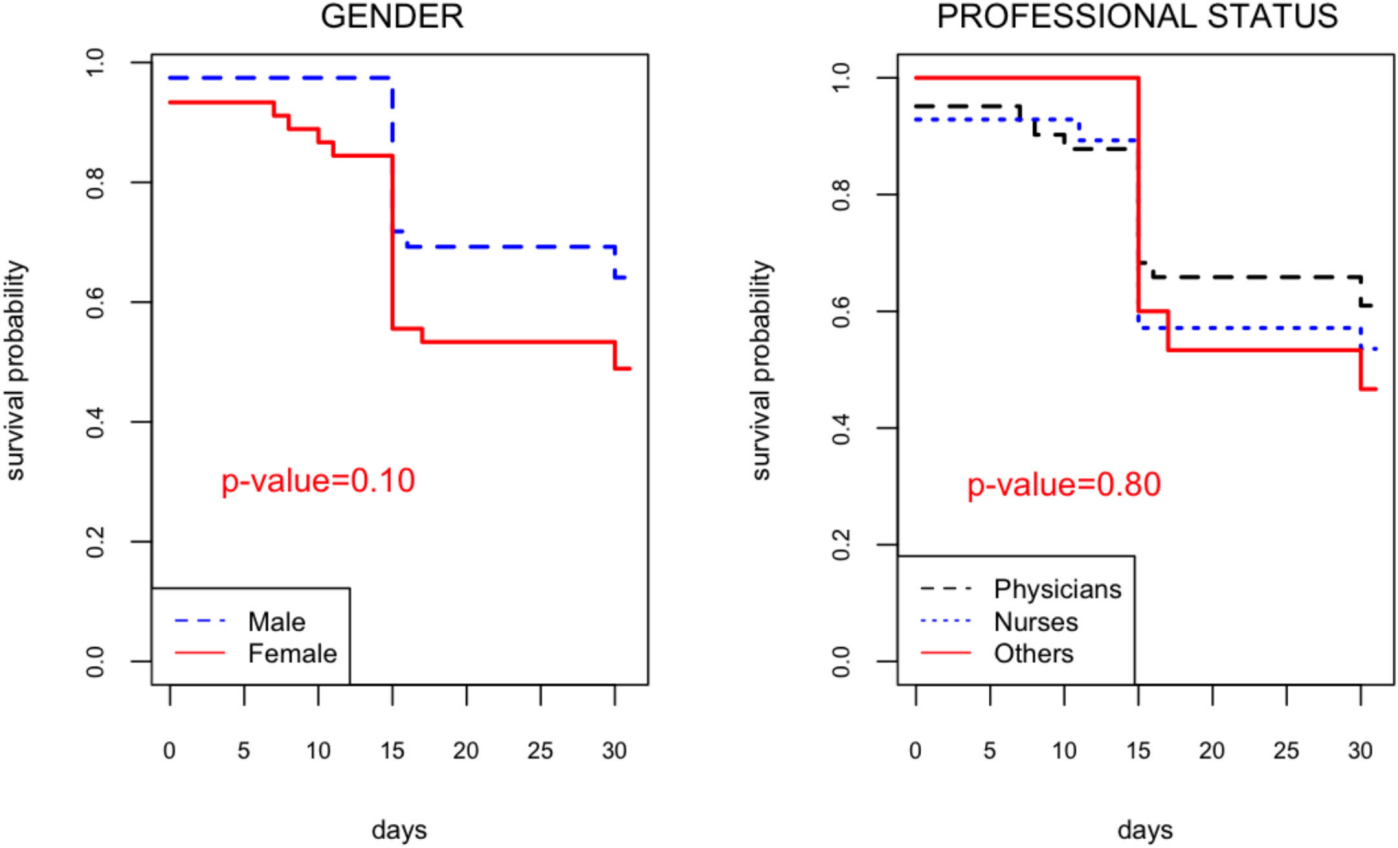
Kaplan–Meier survival estimates by sex and professional status.

Survival analysis (Kaplan–Meier method) showed an attack incidence rate equal to 44 per 100 person*month (95% CI: [32–54]). The incidence was 36 per 100 persons*month (95% CI: [19–49]) for the male and 51 per 100 persons*month (95% CI: [34–64]) (*p*‐value = 0.10). No statistical differences were found regarding sociodemographic characteristics (Figure 1), nor medical history.

**FIGURE 2:**
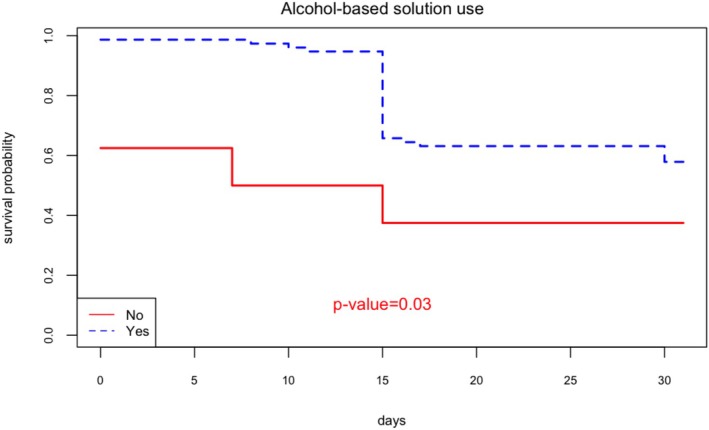
Kaplan–Meier survival estimate for hand cleaning with alcohol‐based solution.

For preventive measures, only alcohol‐based use for hand cleaning was statistically significant (Figure 2). The incidence rate on users was 42 per 100 persons*month (95% CI: [30–52]), whereas for nonusers, it was 63 per 100 persons*month (95% CI: [8–95]) (*p*‐value = 0.03).

The cox regression including the variable with a *p*‐value < 0.20 with a final model with three variables identified two main risk factor (Table 6): female (relative risk = 1.99, *p*‐value < 0.05) and not use of alcohol‐based solution for hand cleaning (RR = 3.5, *p*‐value = 0.013).

**TABLE 6:** Cox regression result (logrank; *p*‐value = 0.01).

	RR [95% CI]	*p*‐value
Gender		
Male	1	
Female	1.99 [1.01–3.99]	0.049
Alcohol‐based use for hand cleaning		
No	1	
Yes	0.28 [0.10–0.76]	0.013
Hand washing after contact with patient		
No	1	
Yes	0.39 [0.14–1.12]	0.076

**TABLE 7:** Description of participants at inclusion and follow‐up step by weeks and by health care facility type.

Weeks	Hospital					Clinic or care center					
	Doctor	Nurses	Others	Total	(%)	Doctor	Nurses	Others	Total	(%)	Total
***At inclusion***											
12		1		**1**	**(50)**	1			**1**	**(50)**	**2**
13	20	8	8	**36**	**(95)**	1		1	**2**	**(5)**	**38**
14	30	17	8	**55**	**(90)**	2	1	3	**6**	**(10)**	**61**
15	3	1	1	**5**	**(100)**						**5**
16		1		**1**	**(100)**						**1**
17	2	15		**17**	**(100)**						**17**
18			5	**5**	**(100)**						**5**
**Total**	**55**	**43**	**22**	**120**	**(93)**	**4**	**1**	**4**	**9**	**(7)**	**129**
***Follow‐up—seronegative at inclusion***											
12				**—**	**—**	1			**1**	**(100)**	**1**
13	11	5	4	**20**	**(91)**	1		1	**2**	**(9)**	**22**
14	21	13	6	**40**	**(89)**	2	1	2	**5**	**(11)**	**45**
15	3	1	1	**5**	**(100)**						**5**
16		1		**1**	**(100)**						**1**
17	2	7		**9**	**(100)**						**9**
18			1	**1**	**(100)**						**1**
**Total**	**37**	**27**	**12**	**76**	**(90)**	**4**	**1**	**3**	**8**	**(10)**	**84**

## 4 Discussion

This prospective cohort study aims to analyze risk factors for COVID‐19 among health workers during the first wave of the SARS‐CoV‐2 epidemic in Niger. It focused on workers who were in contact with the first confirmed cases of SARS‐CoV‐2 in Niamey. The strategy was to follow them for 30 days after contact.

This research was conducted in Niamey, which was the epicenter of the epidemic in Niger. During this time, and despite awareness resulting from the previous Ebola epidemic in the West African region, good clinical practices were not well established in dispensaries and first‐line health structures. In this context, in case of a positive patient attending the ward, all the workers from medical doctors to technicians in laboratories and cleaners could be impacted. The main questions to address were which type of activity was at major risk, and on the other hand, which practices can be protective.

The biological methods used were polymerase chain reaction, which is the gold standard for the diagnosis of infection, and ELISA for serology to demonstrate exposure to SARS‐CoV‐2. However, as it was not possible to practice regular PCR testing for all the workers on a fixed date, this testing was mainly performed when clinical signs were declared. The blood samples for serology were not always accepted by the participants, especially at the end of the study, and 10 results (12%) were missing.

In this study, the country's surveillance capacity to detect the first case and the level of protection during care activities were questionable because of the very high positivity rate at enrollment (35%) and incidence rate during the 1‐month follow‐up. Our result showed a higher incidence rate than similar studies carried out in Cameroun [11] and Madagascar [12] where the incidence rate was around 230 per 1000 persons*month and 100 per 1000 persons*month, respectively. In Egypt, 4% of included HCWs were infected with SARS‐CoV‐2 [13]. In a study conducted from March 2020 to December 2020 in Nabeul, Tunisia, the percentage of SARS‐CoV‐2 positivity among health care personnel was 14% [14]. In Algeria, a descriptive cross‐sectional study conducted from 01/03/2020 to 31/08/2021 recorded a 15% prevalence of COVID‐19 infection [15].

The high rate of contamination of health workers during the first wave of the COVID epidemic underlined not only the poor level of knowledge that they had about the disease and their poor access to individual protection but also the potential contamination in households. During pandemics, HCWs were not only at risk during their professional activities but also in their family and contacts associated with their social life. In Africa, the seroprevalence studies showed wide variations depending on the country and time [16]. In Nigeria and Gabon, the third‐quarter 2020 general population seroprevalence estimations were similar to our results at 25% and 36%, respectively.

Due to the low age of the working population in Niger (median 35 years), most of the HCWs did not harbor comorbidity increasing their risk of severe disease. Although 64% of symptomatic cases were hospitalized, none developed severe symptoms or died.

Our study showed that females were more at risk of infection than males, regardless of their professional status. However, no statistical difference was found according to the functions. The low number of followed HCWs has probably led to a lack of power. Effectively, according to the literature, it is clear that the risk varies by occupation and workstation; a study conducted in the United Kingdom reported a higher seroprevalence among housekeepers (34.5%) and those working in acute medicine (33%) or general internal medicine (30.3%) than in intensive care units welcoming most of the patients (14.8%) [17]. Similar results were reported from Egypt, where 39.6% of positives were nurses, 39.0% were physicians, and 21.4% were nonmedical staff [13]. Frontline health care workers had at least a threefold increased risk of a positive COVID‐19 test and infection, compared with the general community [18].

The lack of information about the practices of invasive procedures (aerosol, etc.), the exact time and care activities in contact with patients for each HCW followed, and the knowledge about the potential exposure in the community were some of the main weaknesses of our study.

Li Ran et al. in Wuhan, China demonstrating that contamination of health care workers is generally due to hand contamination after contact with either patients or fomites [19]. Our results concerning the protective measures showed association between infection risk and use of alcohol‐based solution for hand cleaning and particularly after contact with positive patients. This could be explained by the lack of effective implementation of standard infection prevention and control (IPC) precaution [5].

These results highlight the importance of hand hygiene and alcohol‐based hand cleaning. In Niger, handwashing and water devices were installed in all health facilities by health authorities.

For fighting against infectious transmission risk in hospitals, wards, and other health care facilities, advocacy, training, and implementation of prevention and control measures are of great importance to ensure the protection of HCWs.

## 5 Conclusion

It is important to identify risk factors in the health care setting. This should allow policymakers to develop appropriate strategies to combat this disease. Certainly, both during their activities and through community contacts, HCWs in Niger faced a very high risk of exposure and infection. Concerning risks linked to care activities, policymakers must ensure that emergency and infectious disease services regularly improve training on preventive measures and access to PPE. In the future, issues related to community transmission of HCWs need to be better studied by conducting systematic cohorts of health care workers. This will help to better understand daily practices and identify risks outside of an epidemic or pandemic context.

## Author Contributions

H.L.R., R.J., and V.R. participated in the conception and design of the study. H.L.R., R.J., M.A.M., F.A.A., and V.R. participated in the data analysis. A.S.A.‐K., L.A., F.C.A.S., and I.K. participated in the analysis of the samples in the laboratory. F.A.A., I.I., Z.D.A., B.A.S., G.I.O, A.S., Z.A.A., and A.M. collected field data. H.L.R, R.J., M.A.M., and V.R. drafted the manuscript. G.A.M.B., G.I.O., S.M. analyzes the rough draft. H.L.R., J.R., M.A.M., I.M.L., and A.L. critically reviewed the manuscript. All authors read and approved the final version of this manuscript.

## Conflicts of Interest

The authors declare no conflicts of interest.

## Peer Review

The peer review history for this article is available at https://www.webofscience.com/api/gateway/wos/peer‐review/10.1111/irv.70177.

## Data Availability

The data that support the findings of this study are available from the corresponding author upon reasonable request.
